# Multisession CyberKnife Radiosurgery for Orbital Cavernous Hemangiomas

**DOI:** 10.7759/cureus.12711

**Published:** 2021-01-14

**Authors:** Yusuke Sasaki, Shinichiro Miyazaki, Takanori Fukushima

**Affiliations:** 1 Neurosurgery, Tokyo General Hospital, Tokyo, JPN; 2 Radiation Oncology, Shin-Yurigaoka General Hospital, Kawasaki, JPN; 3 Department of Neurosurgery, Duke University, Durham, USA

**Keywords:** orbital tumor, cavernous hemangioma, cyberknife

## Abstract

Orbital cavernous hemangioma is a rare benign tumor that causes protrusion of the eye-ball and lowers visual acuity. Surgical resection is the first choice of treatment, however, it is challenging due to the anatomical structure of the orbit. Stereotactic radiosurgery (SRS) has become an established alternative treatment for any challenging microsurgery in the skull. Five cases of orbital cavernous hemangioma were successfully treated with multisession CyberKnife (Accuray, Sunnyvale, CA, USA) stereotactic radiotherapy. The tumor volume ranged from 0.2 cc to 11.9 cc, all of which decreased in size after treatment. The radiation prescription dose ranged from 24 Gy to 26.1 Gy with fractions of 5 or 6. Visual acuity was preserved in all cases. Thus, multisession CyberKnife radiotherapy for orbital cavernous hemangioma might be a safe and effective treatment option.

## Introduction

Cavernous hemangiomas in the orbit occur in 9% of orbital tumors and 3% of all intracranial cavernous hemangiomas [1]. Cavernous hemangioma often presents in middle-aged patients and is common in women because of the effects of sex hormones on its pathogenesis and growth [2]. An intra-axial cavernous hemangioma may cause symptomatic intracranial hemorrhage. An extra-axial cavernous hemangioma rarely presents hemorrhage, but commonly presents cranial nerve deficits and intracranial mass effect. Treatment strategies such as surgical resection, conventional radiotherapy, and stereotactic body radiation therapy (SBRT) are chosen according to location and symptoms. The first choice of treatment is surgical resection; however, this is challenging due to the complex structure of the orbit with its small vessels and nerves.

CyberKnife (Accuray, Inc., Sunnyvale, CA) delivers stereotactic radiosurgery (SRS) and SBRT with robotic precision. It is non-invasive compared to standard surgery, and thus, a promising treatment option for orbital cavernous hemangioma.

## Case presentation

Three male and two female patients were treated with multisession CyberKnife radiotherapy at Shin-yurigaoka General Hospital between 2012 and 2018 as shown in Table 1. All five patients were symptomatic with exophthalmos and impaired vision. The median tumor volume was 2.6 cc (ranged 0.4 - 11.9 cc). The median prescription dose was 25 Gy (ranged 24 - 26.1 Gy). All treatments were performed on consecutive days and completed within a week with five or six fractions.

**Table 1:** Five cases of orbital cavernous hemangioma CR: complete response, PR: partial response.

	Sex	Age	Symptoms	Location	Volume (ml)	Marginal dose (Gy)	Fraction	F/U (M0)	Result
1	F	56	Visual loss	Left	11.9	26.1	6	84	PR
2	M	66	Visual loss	Right	0.4	25	5	72	CR
3	M	55	Lack of vision	Right	5.5	24	5	60	PR
4	F	52	Visual loss	Right	2.6	24	5	48	PR
5	M	35	None	Right	2.8	25	5	27	PR

Gadolinium-enhanced brain MRI and contrast brain CT were performed for follow-up evaluation. The follow-up period was 3 to 84 months. Tumor volume was remarkably decreased in all cases. Symptoms of all patients improved, and none presented neither adverse effects nor new symptoms. Visual field was preserved, and visual acuity improved for all patients.

**Figure 1:**
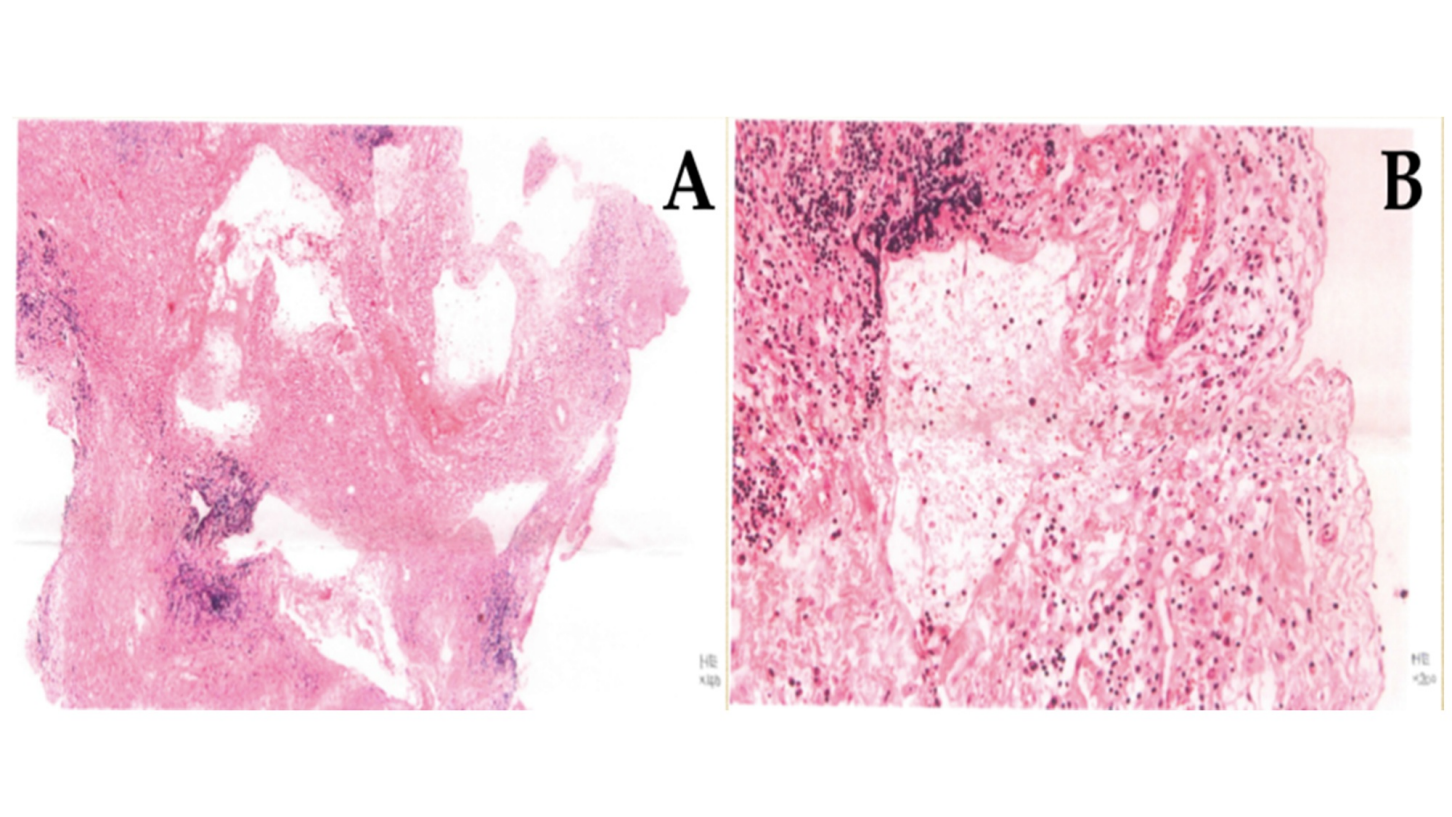
Pathological findings of case 1 Hematoxylin-eosin staining revealed proliferation of small vessels and infiltration of lymphocytes. (A) Low power field, ×4 and (B) high power field, ×40.

**Figure 2:**
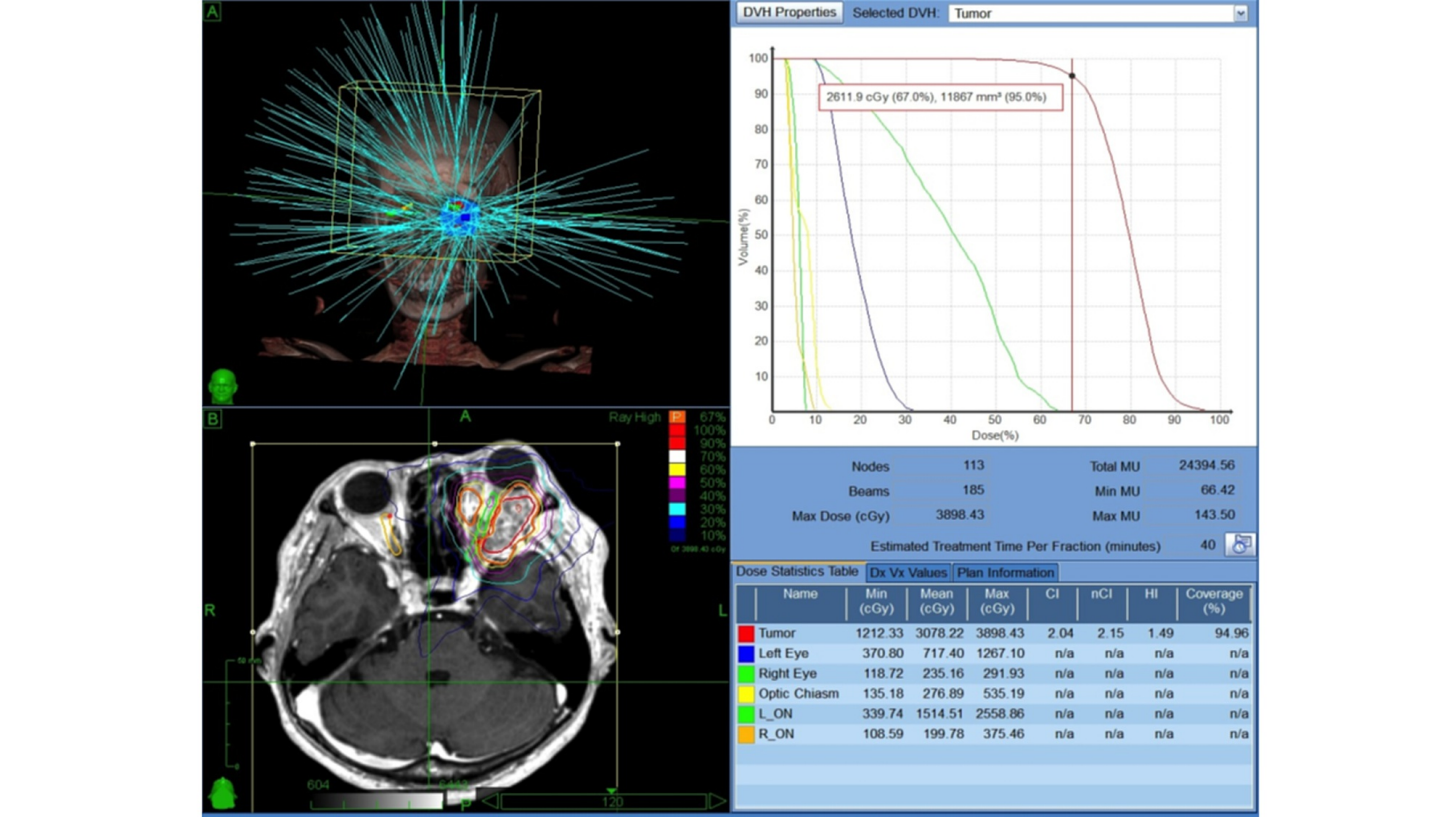
CyberKnife treatment plan for case 1 The tumor volume was 11.9 cc, the prescription dose was 26.1 Gy, and prescription isodose was 67%.

**Figure 3:**
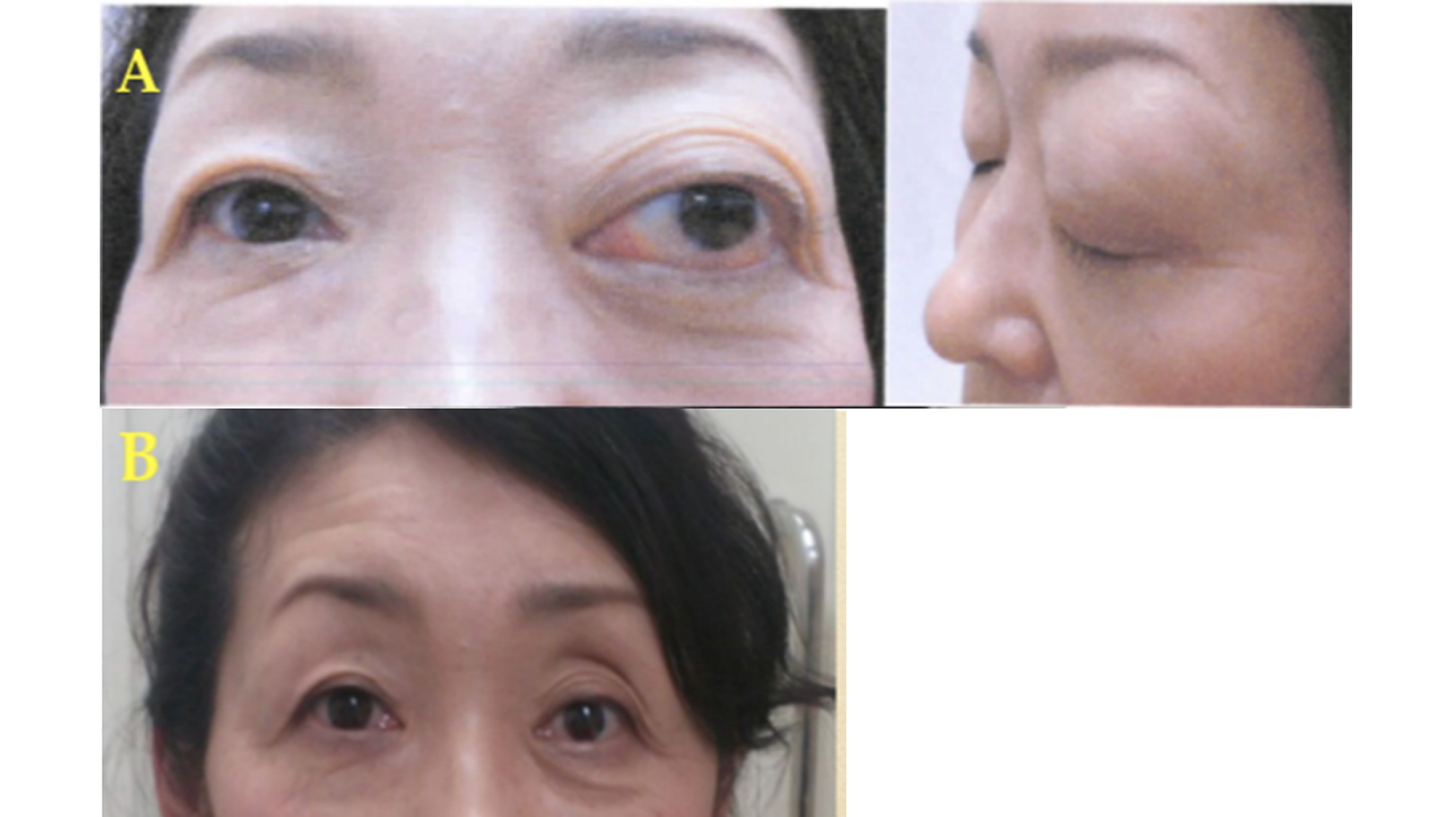
Facial appearance of case 1 Exophthalmos was remarkable before treatment (A), which resolved after treatment (B).

The most prominent case is case 1 in Table 1. Case 1 is a 56-year-old woman who visited an ophthalmologist for visual disturbances and was diagnosed with asthenopia. However, her vision deteriorated over three months and she presented with exophthalmos. Brain MRI revealed an orbital tumor that was suggestive of orbital cavernous hemangioma. Biopsy and pathological examination confirmed that the tumor was a cavernous hemangioma (Figure 1). CyberKnife SRS was performed for tumor volume of 11.9 cc with a prescription dose of 26.1 Gy in six fractions (Figure 2). One year after treatment, the patient’s symptoms remarkably improved and a brain MRI revealed tumor decreased in size (Figure 3).

**Figure 4:**
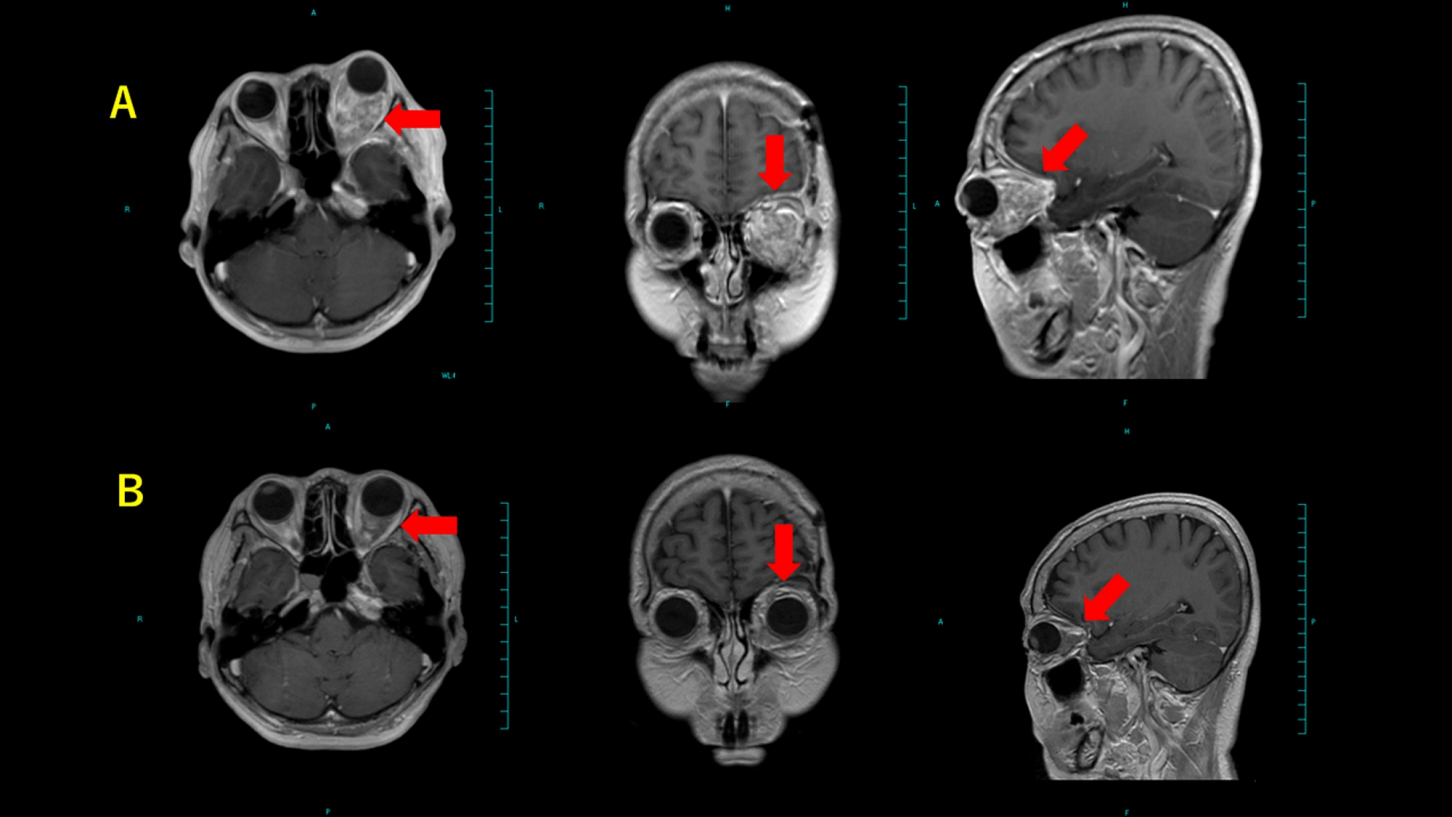
Brain MRI of case 1 (A) Before treatment and (B) 84 months after treatment. Red arrows denote tumor.

**Figure 5:**
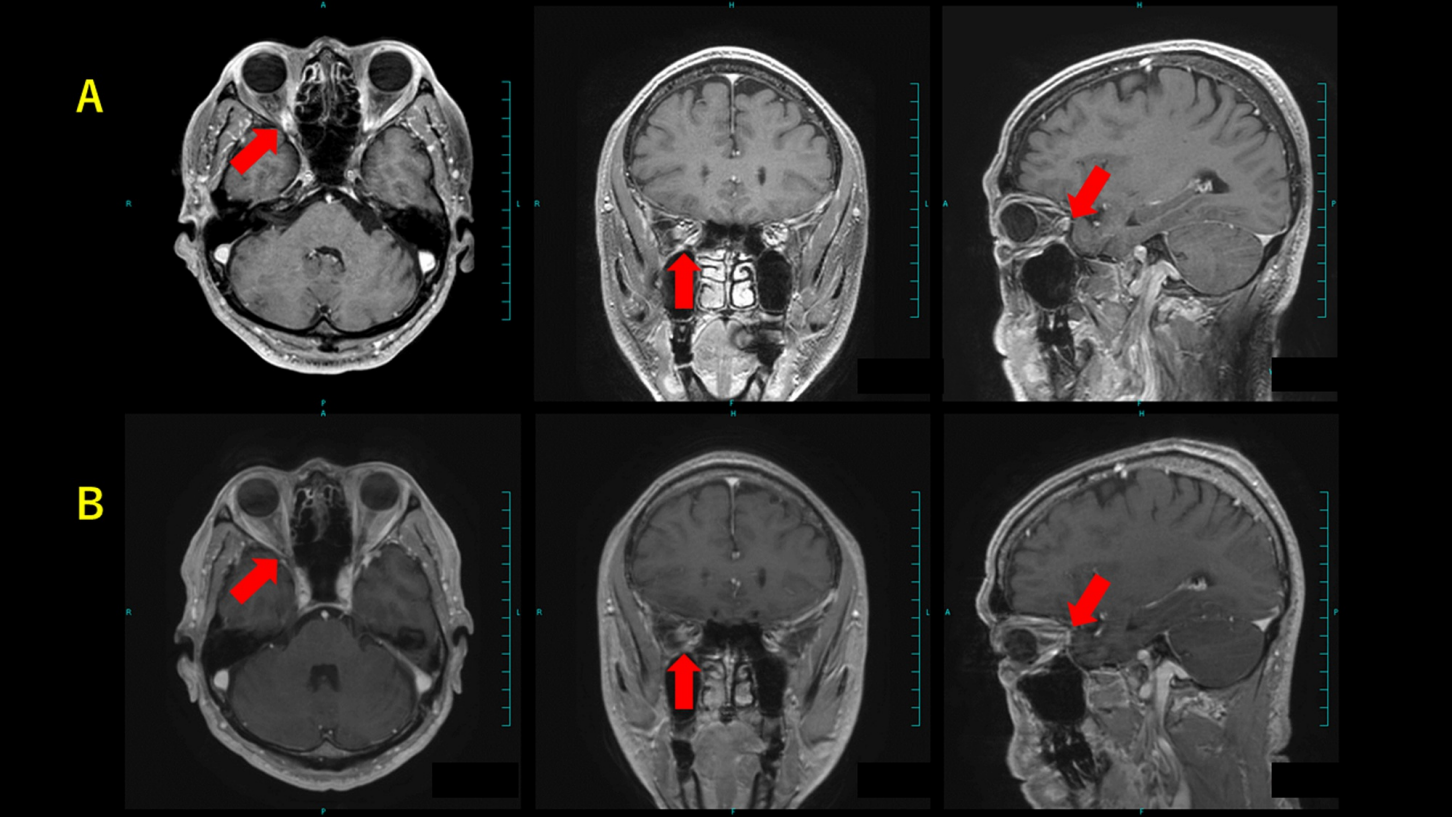
Brain MRI of case 2 Case 2 had decreased visual acuity and visual field narrowing due to the intra-optic canal tumor. (A) Before treatment and (B) 72 months after treatment. Red arrows denote tumor. The tumor size decreased; visual acuity and visual field were recovered.

**Figure 6:**
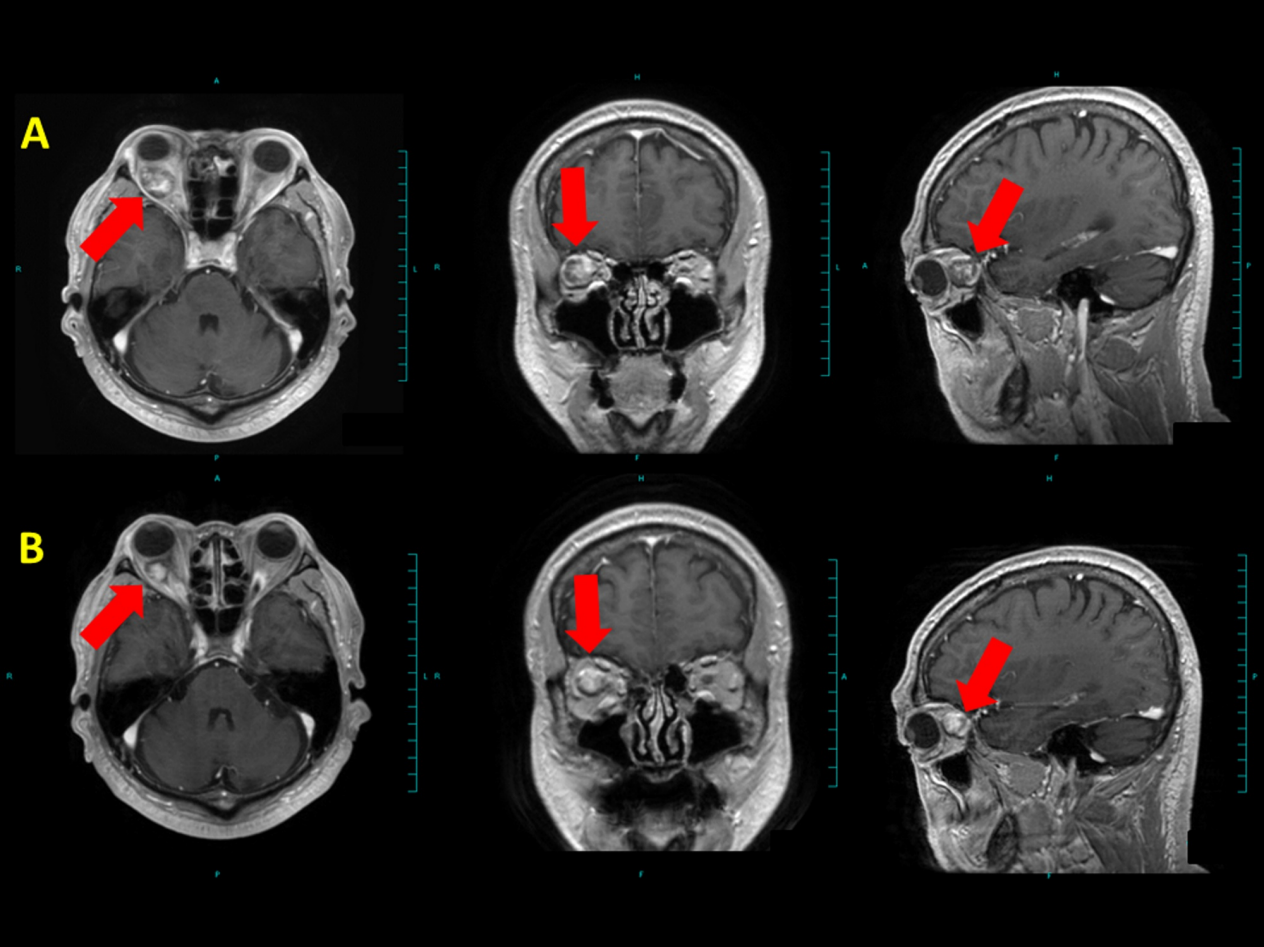
Brain MRI of case 3 Case 3 had decreased visual acuity and exophthalmos. (A) Before treatment and (B) 18 months after treatment. Red arrows denote tumor. The tumor decreased in size; visual acuity and exophthalmos were recovered.

**Figure 7:**
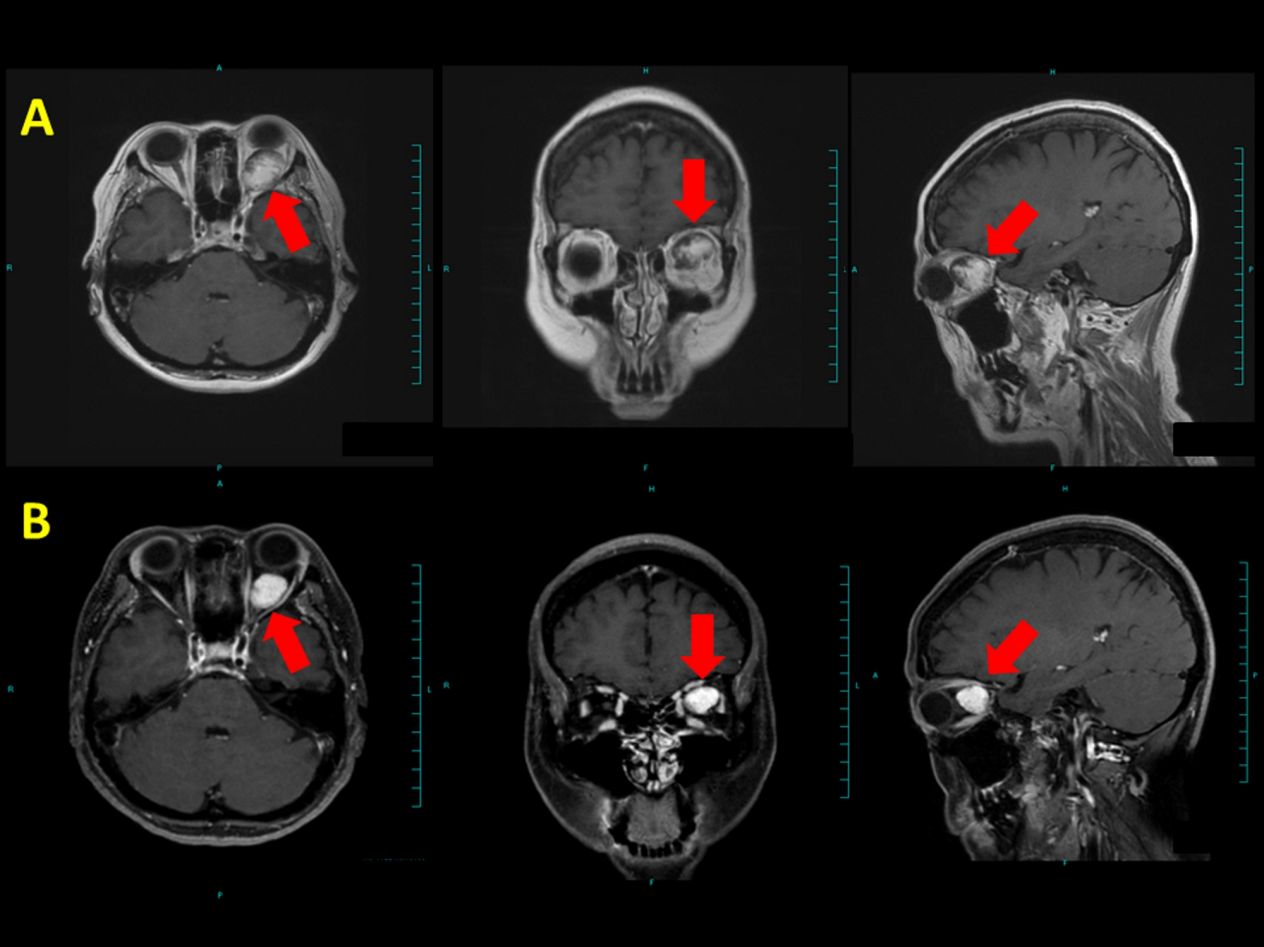
Brain MRI of case 4 Case 4 had extremely decreased visual acuity and exophthalmos. (A) Before treatment and (B) 18 months after treatment. Red arrows denote tumor. The tumor decreased in size; visual acuity and exophthalmos were recovered.

**Figure 8:**
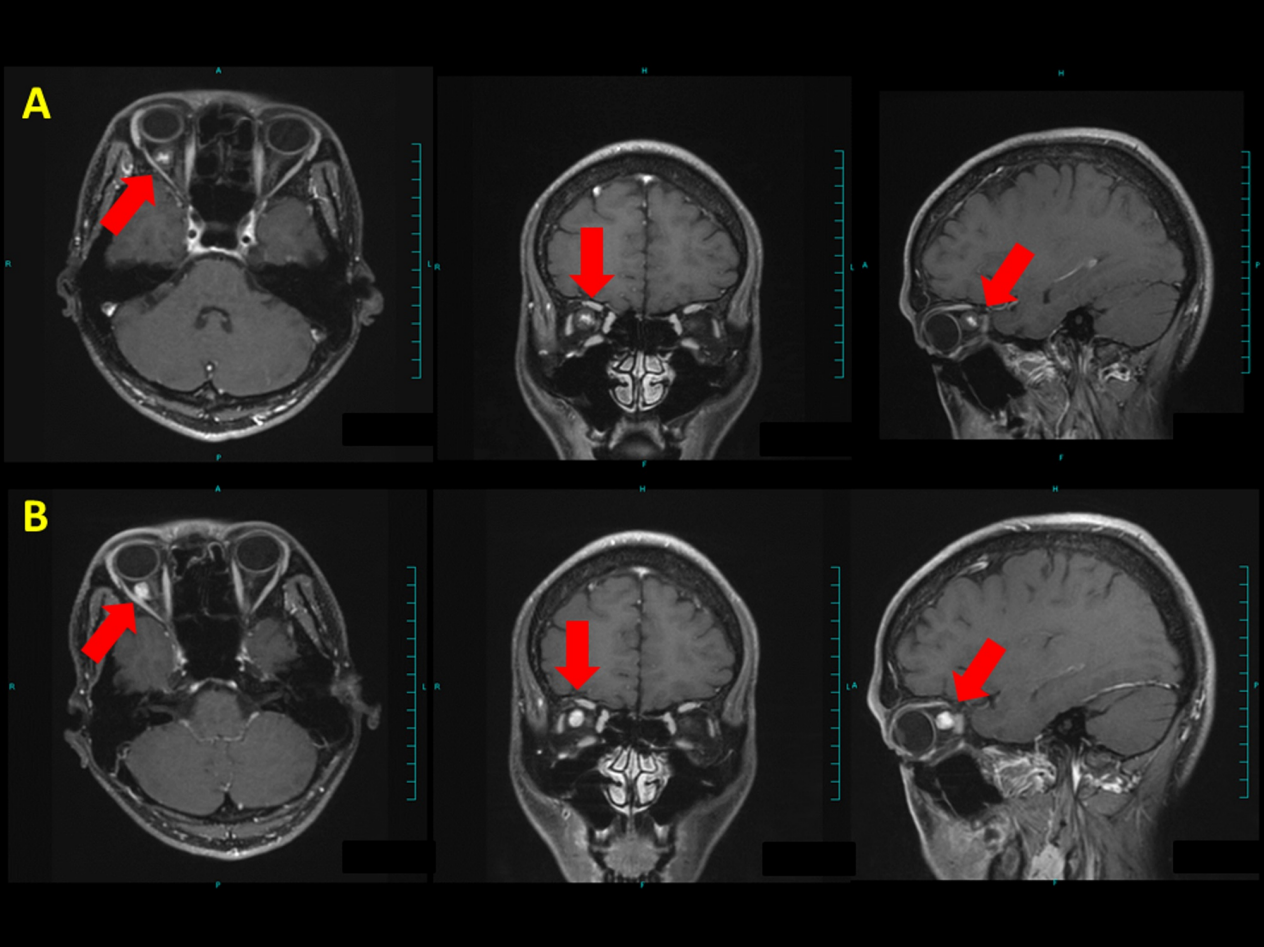
Brain MRI of case 5 (A) Before treatment and (B) 8 months after treatment. Red arrows denote tumor. The tumor decreased in size.

Gadolinium-enhanced brain MRI before and after treatment are shown in Figures 4 - 8. It is noteworthy that tumors remarkably decreased in size.

## Discussion

Cavernous hemangiomas occurring within the cavernous sinus, including sagittal sinus, extradural, and orbit, differ from intra-axial cavernous malformation [3]. In the intra-orbital area, the most common tumors in senior patients are cavernous hemangiomas [4]. The phenotype of an orbital tumor can be estimated by the patient’s age and location of the tumor. An orbital cavernous hemangioma may require treatment for longer maximal linear dimension, male gender, or extra-orbital location [5]. Management of this tumor ranges from periodic observation for small asymptomatic lesions to surgical excision for large symptomatic tumors [6]. A biopsy is preferred to confirm the diagnosis; however, this may cause critical bleeding as hemangioma is rich in blood vessels.

Boari et al. suggested immediate surgical excision of orbital cavernous hemangioma causing visual deficits, as an extended period of visual deterioration minimizes the chance for recovery [6]. They also stated that visual outcome in orbital cavernous surgery is strongly affected by the planning of the correct surgical approach in relation to the location of the tumor and by the dissecting technique used to remove the tumor [6].

There is a consensus that orbital cavernous hemangiomas are extra-axial lesions and exhibit different clinical behavior from intra-axial cavernous malformations [3]. Cavernous sinus or orbital cavernous hemangioma is sensitive to radiation compared to intra-axial cavernous malformations [3]. Thompson et al. reported treatment with Gamma Knife radiation therapy for hemangiomas of cavernous sinus and orbit has been successful and is associated with a mean reduction of 54% in tumor volume [7]. Khan et al. reported that symptomatic improvement occurred in all patients with cavernous or orbital hemangiomas with the treatment of Gamma Knife radiation, and no patient experienced adverse radiation effects [8]. SRS avoids complications associated with embolization, biopsy, and attempted microsurgical resection [8]. Thus, Gamma Knife radiosurgery (GKS) for extra-dural cavernous hemangioma has gained popularity. GKS is usually performed in a single session. The risk of traditional single session GKS is increased because the orbital lesion is adjacent to radiation-sensitive critical structures. However, Kim et al. reported that multisession GKS proved to be an effective and safe management strategy for orbital apex tumors [9]. Orbital apex tumors included cavernous hemangiomas (eight cases), meningioma (eight cases), and schwannoma (seven cases). All patients were treated with four sessions of GKS with a 12-hour interval. Significant tumor shrinkage was observed in 17 patients (73.9%), with a 53.9% mean tumor volume reduction rate [9].

Radiation fractionation is essential in radiation therapy. Hirshbein et al. report that despite aggressive irradiation and large fraction size, staged radiosurgical ablation with the CyberKnife system appeared to be safe, especially as measured by visual field preservation and visual acuity [10]. The optic pathway should not receive radiation of more than 10 Gy. Calandriello et al. irradiated the optic nerve of chiasm with more than 8 to 10 Gy in a single fraction and showed a risk of visual injury [11]. With CyberKnife, it was possible to keep a single-session dose to the visual pathways to less than 5 Gy [11]. The small number of fractions with CyberKnife was effective to minimize visual complications [12]. In this study, radiation doses were delivered in five or six fractions, and no patients lost further visual field or visual acuity. All tumors remarkably decreased in size. Using more than five radiation fractions, the anterior optic pathway was protected. The maximal dose to the optic nerve was 17.4 Gy to 26.6 Gy over five to six fractions, and the maximal dose to optic chiasm was 1.4 Gy to 5.4 Gy over five to six fractions.

The patients reported that their visual acuity had been improved and no objective measures were available to them. This is a limitation of this research.

## Conclusions

Our case report demonstrated that multisession CyberKnife radiosurgery might be safe and effective for orbital cavernous hemangioma. Multisession SRS for orbital cavernous hemangioma may thus become first treatment option. Longer-term follow-up is anticipated to further confirm efficacy and safety of this treatment.
